# Effect of Fe and Si Content on Microstructure, Mechanical Properties, and Corrosion Resistance of 7050 Alloy

**DOI:** 10.3390/ma19010135

**Published:** 2025-12-30

**Authors:** Changlin Li, Wei Zhao, Tingrui Zhang, Xiwu Li, Zhicheng Liu, Ying Li, Lizhen Yan, Pengfei Xu, Kai Wen, Yongan Zhang, Zhihui Li, Baiqing Xiong

**Affiliations:** 1 State Key Laboratory of Nonferrous Structural Materials, China GRINM Group Co., Ltd., Beijing 100088, China; lichanglin@grinm.com (C.L.); 13926801390@163.com (T.Z.); liying@grinm.com (Y.L.); yanlizhen@grinm.com (L.Y.); wenkai@grinm.com (K.W.); zhangyongan@grinm.com (Y.Z.); lizhihui@grinm.com (Z.L.); xiongbq@grinm.com (B.X.); 2 GRIMAT Engineering Institute Co., Ltd., Beijing 101407, China; 3 School of Rare Earth Industry, Inner Mongolia University of Science and Technology, Baotou 014010, China; 4 Baotou Aluminum Co., Ltd., Baotou 014046, China; 5 General Research Institute for Nonferrous Metals, Beijing 100088, China

**Keywords:** 7050 aluminum alloy, Fe and Si impurities, microstructure, mechanical properties, corrosion resistance

## Abstract

In this work, the effect of Fe and Si content on microstructure, mechanical properties, and corrosion resistance of 7050 alloy was systematically investigated by room temperature tensile, fracture toughness, and exfoliation corrosion tests, complemented by microstructural characterization through SEM and TEM. The results demonstrate that the impurity elements Fe and Si induce the formation of insoluble Fe-rich phases and Mg_2_Si phases in the alloy, respectively. The coexistence of Fe and Si leads to a severe synergistic deterioration effect on mechanical properties. Furthermore, the study reveals that Si has a more profound negative impact on mechanical properties than Fe. While Fe primarily reduces ductility and fracture toughness by initiating microcracks through Fe-rich phases with minimal effect on strength, Si not only forms brittle Mg_2_Si phases that impair toughness but also significantly depletes the Mg content in the matrix, thereby reducing the quantity of strengthening phases. This results in a comprehensive and severe decline in strength, plasticity, and toughness. In addition, Fe and Si impurities markedly degrade the exfoliation corrosion resistance of the alloy.

## 1. Introduction

Al-Zn-Mg-Cu high-strength aluminum alloys, particularly the 7050 alloy, have emerged as essential materials for aerospace structural components owing to their outstanding specific strength, fracture toughness, and fatigue resistance [1,2,3,4]. With the continued advancement of green manufacturing and sustainable development initiatives [5], the use of recycled aluminum has become increasingly prominent in the aluminum alloy industry [6]. However, during repeated recycling of aluminum materials, impurity elements such as Fe and Si tend to accumulate [7,8]. These elements stem mainly from coatings, inclusions, and admixed metals in aluminum scrap, and their concentrations in recycled aluminum are generally much higher than in primary aluminum. This poses significant challenges to preserving the superior overall performance of aluminum alloys

In 7xxx series aluminum alloys, the solubility of Fe and Si in the Al matrix is extremely low. During melting, solidification, and subsequent heat treatment, they tend to form various coarse, hard, and brittle intermetallic compound phases with aluminum and other alloying elements [9,10,11]. Research indicates that common Fe-rich phases include Al_3_Fe [12], Al_7_Cu_2_Fe [13,14], and Al_6_(FeMn) [15], while Si-rich phases may form Mg_2_Si. Furthermore, when Fe and Si coexist, complex phases such as α-AlFeSi and β-AlFeSi are more likely to form [16,17,18].

The Al_7_Cu_2_Fe phase is a common Fe-rich intermetallic phase found in 7xxx series alloys, with an orthorhombic crystal structure. It typically presents as coarse or blocky-shaped particles. These coarse Al_7_Cu_2_Fe particles are hard and brittle [19,20]. Consequently, under mechanical loading, it is prone to cracking at the interface with the aluminum matrix, serving as a site for microcrack initiation. As an impurity in 7xxx series alloys, Si preferentially binds with Mg during solidification, forming stable but non-strengthening Mg_2_Si phases [21,22]. These coarse particles act as stress concentrators. Furthermore, Si excessively depletes the available Mg, thereby hindering the formation of the key strengthening MgZn_2_ phase. These effects collectively lead to a significant deterioration in mechanical properties and corrosion resistance.

The β-AlFeSi phase (Al_5_FeSi) possesses a monoclinic crystal structure and typically exhibits a plate-like or needle-like morphology [23,24,25]. It is considered the most detrimental phase to mechanical properties. These plate-like β-AlFeSi particles act as inherent micro-cracks within the matrix. Under external loading, their sharp tips induce severe stress concentration, making them preferred sites for micro-crack initiation. They significantly disrupt the continuity of the aluminum matrix, hinder the coordinated movement of dislocations, and lead to a sharp decline in elongation and toughness. In contrast, the α-AlFeSi phase (Al_8_Fe_2_Si or Al_15_(Fe,Mn)_3_Si_2_) usually crystallizes in a cubic structure and commonly appears as clusters of interconnected “Chinese script” morphology [5,26]. This morphology lacks sharp edges, resulting in a much less detrimental effect on alloy properties compared to the β-AlFeSi phase [27].

Although existing research has extensively investigated impurity phases in aluminum alloys and widely recognized the detrimental effects of Fe and Si elements on alloy performance, industrial practice has thus often favored the use of high-purity aluminum ingots for production to control impurity content (e.g., Fe ≤ 0.15 wt.%, Si ≤ 0.12 wt.%) at the source [28,29,30]. However, in the current context of vigorously promoting sustainable manufacturing with recycled raw materials, Fe and Si are the most typical and difficult-to-remove impurity elements in recycled aluminum. Therefore, it is particularly important to systematically investigate the influence of variations in Fe and Si content and their coupling effects on the microstructure and properties of alloys. This study aims to provide empirical evidence and theoretical insights, thereby laying a foundation for scientifically establishing control ranges for Fe and Si in 7050 alloy.

This study systematically designs and prepares a series of 7050 alloys with varied Fe and Si contents to investigate their influence on microstructure, mechanical properties, and corrosion resistance. The objective is to provide clear theoretical foundations and data support for optimizing the composition and ensuring quality control of high-performance 7050 alloys.

## 2. Materials and Methods

**Figure 1:**
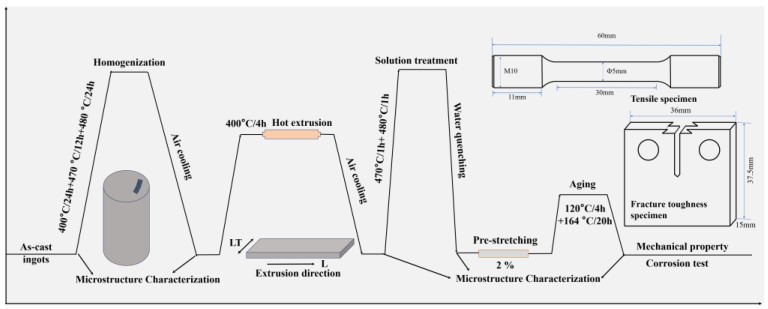
Schematic diagram of the experimental route.

Figure 1 shows the schematic diagram of the experimental route. The 7050 aluminum alloy ingots used for subsequent hot extrusion were fabricated by melting and casting route. Pure aluminum was first melted, followed by the addition of pure Cu and Zn at 720 °C. The temperature was then raised to 750 °C and held for 30 min. Afterwards, the melt was cooled down to 720 °C, at which point pure Mg and a Mg-30%Zr master alloy were added. After thorough stirring, the melt was refined using argon gas and allowed to settle for 10 min. Finally, it was poured into a water-cooled copper mold to obtain the cast ingot. After the ingot was cooled to room temperature, samples were taken for chemical composition analysis. The contents of the elements were determined using inductively coupled plasma atomic emission spectroscopy (ICP-AES, PerkinElmer Avio 500, Singapore). Debris were drilled from the ingot at the quarter-radius position, dissolved in an HCl/HNO_3_ mixed acid, and prepared as test solutions. Calibration was performed using matrix-matched standard solutions. Each sample was measured three times, with the measurement uncertainty for the main alloying elements being within ±0.1 wt.% and that for the microalloying elements within ±0.05 wt.%. The actual composition is listed in Table 1. To eliminate dendritic segregation and achieve a more homogeneous microstructure, the ingots underwent a three-step homogenization treatment (400 °C/24 h + 470 °C/12 h +480 °C/24 h). The temperature of 400 °C was set to promote the precipitation of Al_3_Zr particles, 470 °C was aimed at dissolving the AlZnMgCu phase back into the matrix, and 480 °C was intended to dissolve both the Al_2_CuMg phase and the Al_2_Cu phase. The ingot was hot-extruded into a rectangular plate measuring 63 mm × 16 mm using a 1750 t extrusion press. The extrusion speed was set at 0.5 mm/s. The deformation temperature was maintained at approximately 400 °C. Based on DSC analysis results and previous studies on 7xxx series aluminum alloys [31,32], the extruded plates underwent a double-stage solution treatment at 470 °C for 1 h followed by 480 °C for 1 h, and were then immediately water-quenched. To further regulate the microstructure and properties, a 2% pre-stretching deformation was applied to the samples, followed by artificial aging treatment at 120 °C for 4 h and 164 °C for 20 h.

Tensile properties were evaluated using an MTS CMC 4304 universal testing machine (Eden Prairie, MN, USA) with a constant crosshead speed of 2 mm/min. The plane-strain fracture toughness (KIC) tests were carried out on an MTS 370.10 Landmark servo-hydraulic testing system in accordance with ASTM E399 [33]. Additionally, exfoliation corrosion (EXCO) behavior was examined following the procedure specified in the standard GB/T 22639-2022 [34].

Observations of the metallographic structure were carried out employing a Zeiss Axiovert 200 MAT (Oberkochen, Germany) model optical microscope. The microstructural analysis was conducted using a JEOL JSM 7900F field-emission scanning electron microscope (SEM) (Tokyo, Japan), equipped with an energy-dispersive spectroscopy (EDS) module. Observations were carried out at an accelerating voltage of 20 kV. Precipitate characterization was performed via transmission electron microscopy (TEM) on an FEI Tecnai G2 F20 field-emission TEM (Hillsboro, OR, USA) operating at 200 kV.

## 3. Results and Discussion

### 3.1. Microstructure Characteristics of As-Cast Alloys

**Figure 2:**
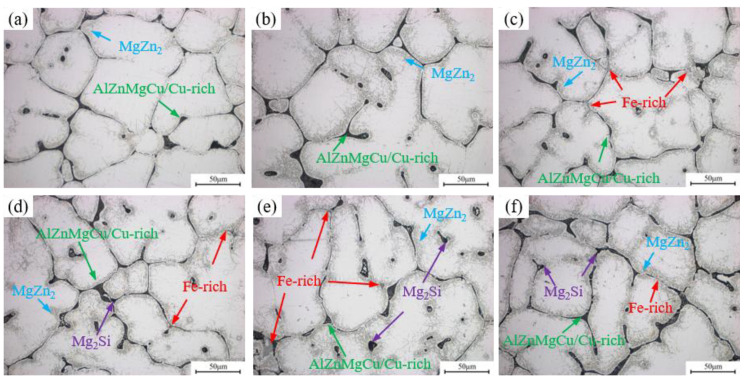
OM images of as-cast alloys: (**a**) Base alloy; (**b**) 0.03Fe 0.02Si; (**c**) 0.15Fe 0.02Si; (**d**) 0.12Fe 0.08Si; (**e**) 0.15Fe 0.12Si; (**f**) 0.03Fe 0.12Si.

The OM images of as-cast alloys with different Fe and Si contents are shown in Figure 2. The as-cast microstructure of all alloys exhibits a typical dendritic network structure with severe dendritic segregation. The bright regions in the images represent the α-Al matrix, while the black network and intragranular ellipsoidal structures constitute the primary solidified eutectic phase formed during non-equilibrium solidification. Based on the research findings of Yin [32], eutectic phases are mainly divided into two types. The first type consists of re-soluble second phases, including black reticular or blocky AlZnMgCu (Mg(Zn,Cu,Al)_2_ phase), Cu-rich eutectic phases (Al_2_Cu, Al_2_CuMg phase), and MgZn_2_ phase precipitated during solidification near grain boundaries. The other type consists of insoluble secondary phases, primarily gray Fe-rich phases (mainly comprising Al_7_Cu_2_Fe, Al_6_Fe, and α-AlFeSi phases) and black Mg_2_Si phases. As the Fe content increases, particularly in the 0.15Fe 0.02Si alloy, the Fe-rich phase significantly increases. When the Si content rises to 0.08 wt.%, the primary Mg_2_Si phase appears in the alloy. The XRD results are shown in Supplementary Figure S1.

**Figure 3:**
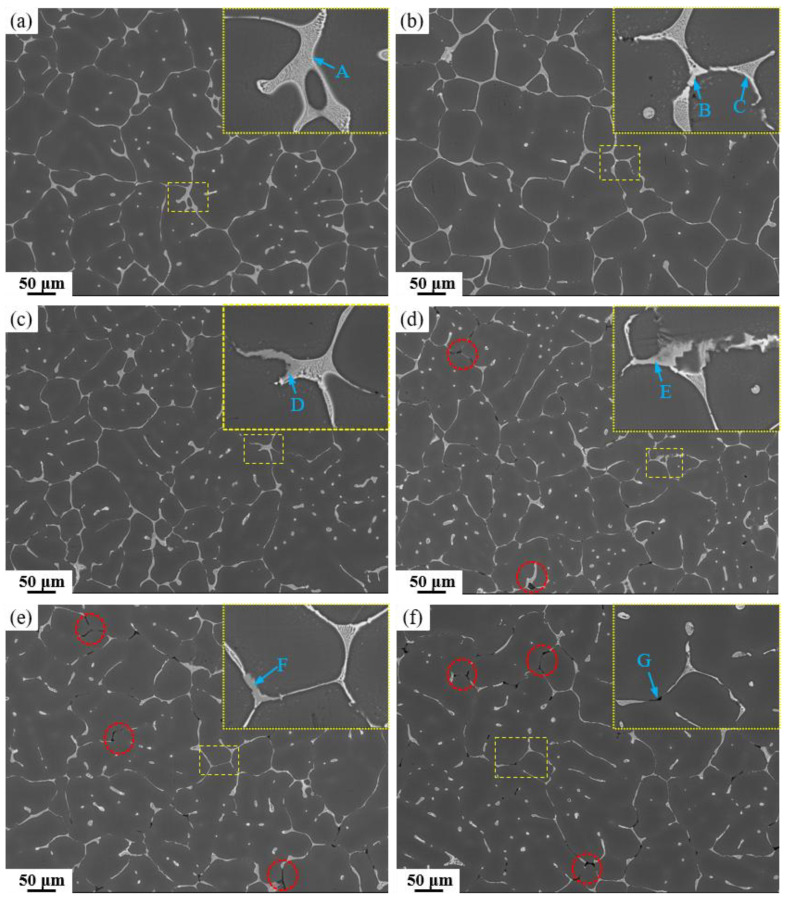
SEM images of as-cast alloys: (**a**) Base alloy; (**b**) 0.03Fe 0.02Si; (**c**) 0.15Fe 0.02Si; (**d**) 0.12Fe 0.08Si; (**e**) 0.15Fe 0.12Si; (**f**) 0.03Fe 0.12Si.

Figure 3 shows low magnification and locally magnified (yellow boxes) SEM images of as-cast alloys with different Fe and Si contents. Table 2 presents the corresponding EDS analysis results for marked locations (the energy-dispersive spectrum can be found in Supplementary Figure S2). It can be observed that the lattice-like secondary phase primarily consists of the AlZnMgCu quaternary eutectic phase, and minor amounts of Al_2_Cu phase are also present. In the 0.15 Fe alloys, the gray Fe-rich phase appears. Analysis of the EDS results indicates that the Fe-rich phase has a low Cu content, primarily consisting of Al_6_Fe. Additionally, Figure 3d,e reveals the presence of black Mg_2_Si phases, with a significantly higher content of Mg_2_Si phases (red circles) observed in the high Si-containing alloys.

**Figure 4:**
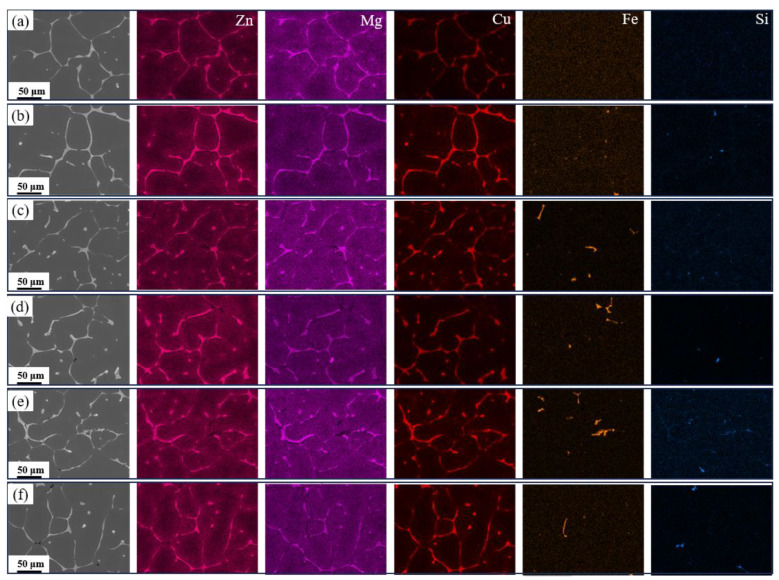
Elemental mapping images of the as-cast alloy: (**a**) Base alloy; (**b**) 0.03Fe 0.02Si; (**c**) 0.15Fe 0.02Si; (**d**) 0.12Fe 0.08Si; (**e**) 0.15Fe 0.12Si; (**f**) 0.03Fe 0.12Si.

To further identify the elemental distribution in the alloys, Figure 4 shows elemental mapping images of as-cast alloys with varying Fe and Si contents. It can be observed that the eutectic phase primarily contains Mg, Zn, and Cu elements, further confirming that the AlZnMgCu quaternary eutectic phase dominates in the alloy. Additionally, no significant Fe or Si segregation was detected in the base alloy. As Fe content increased, Fe elements segregated to form Fe-rich phases. As Si content further increased, both Fe and Si elements co-segregated to form AlFeSi and Mg_2_Si phases.

**Figure 5:**
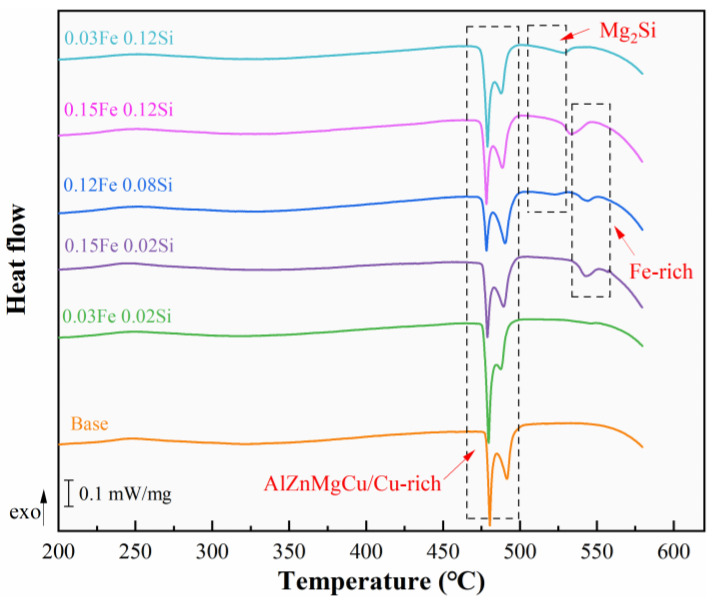
DSC curve of as-cast alloy.

Figure 5 presents the DSC curves of as-cast alloys. Three major categories of endothermic peaks are detected in the alloys. In high Fe-containing alloys, an endothermic peak corresponding to the Fe-rich phase appears near 540 °C, while in high Si-containing alloys, an endothermic peak associated with the Mg_2_Si phase appears near 520 °C. The 0.15Fe 0.12Si alloy, with higher Fe and Si contents, exhibits significant amounts of both Fe-rich and Mg_2_Si phases, causing the two endothermic peaks to be essentially combined. All alloys exhibit an endothermic peak for the re-soluble phase between 470 °C and 500 °C. The phase with the lower initial melting temperature is the AlZnMgCu quaternary eutectic phase, while the phase with the higher initial melting temperature is the Cu-rich phase (Al_2_Cu or Al_2_CuMg). The DSC curve results are consistent with the previous microstructure results.

**Figure 6:**
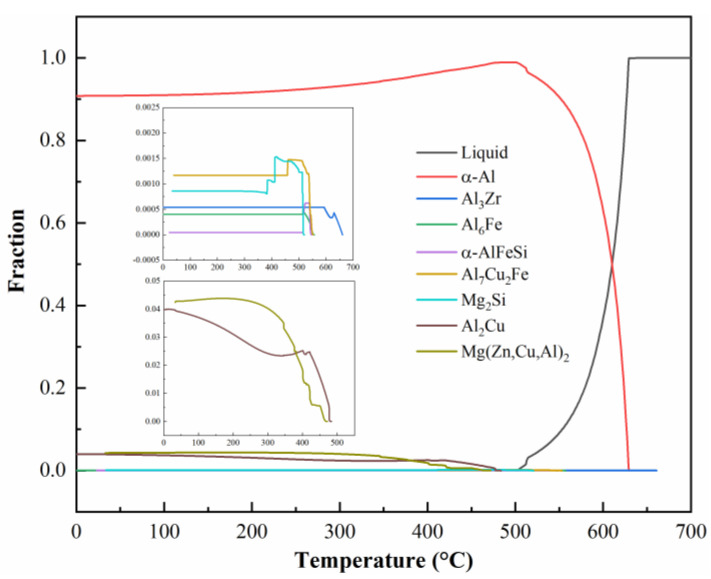
Solidification curves of 0.15Fe 0.12Si alloy.

The solidification pathway of the 0.15Fe 0.12Si alloy during cooling from 700 °C to room temperature was simulated using Pandat 2022 software, as presented in Figure 6. Upon cooling, primary Al_3_Zr particles start to precipitate around 660 °C, thus providing heterogeneous nucleation sites for the subsequent α-Al matrix. At 630 °C, the α-Al phase begins to form. With continued cooling, Al_6_Fe, α-AlFeSi, and Al_7_Cu_2_Fe phases precipitate sequentially starting at 550 °C. By 520 °C, the Mg_2_Si phase begins to form. As the temperature further decreases to 490 °C, substantial precipitation of Al_2_Cu and AlZnMgCu phases occurs progressively. Overall, the insoluble Fe-rich phase exhibits a higher content compared to the Mg_2_Si phase, which aligns well with the earlier microstructural observations.

Comprehensive analysis reveals that the addition of Fe and Si elements significantly alters the solidification pathway and final phase constituents of the experimental alloy. The types of eutectic phases observed in this study, such as the re-soluble AlZnMgCu and Al_2_Cu phases, and the insoluble Fe-rich and Mg_2_Si phases, are consistent with previous reports [29,31], confirming the universality of this secondary phase classification in this alloy system. However, by systematically varying the Fe and Si content, this work further quantitatively elucidates their competitive roles during solidification: an increase in Fe prominently promotes the formation of Fe-rich phases such as Al_6_Fe and Al_7_Cu_2_Fe, while Si, besides partially dissolving in the α-Al matrix and participating in the formation of the α-AlFeSi phase, primarily acts to induce the precipitation of the Mg_2_Si phase.

We obtained mutually corroborating results and mechanistic explanations through DSC analysis and Pandat thermodynamic simulations. The simulation results accurately predict the precipitation sequence where Fe-rich phases (Al_6_Fe, α-AlFeSi, Al_7_Cu_2_Fe) form prior to the Mg_2_Si phase. This sequence aligns perfectly with the DSC curves, where the endothermic peak corresponding to the Fe-rich phases appears at a higher temperature range (~540 °C). Particularly in the 0.15Fe 0.12Si alloy, the substantial formation of both Fe-rich and Mg_2_Si phases leads to the convergence of their precipitation temperature intervals, causing the overlap of their respective endothermic peaks in the DSC curve. This experimental observation is successfully replicated by the thermodynamic simulation. Ultimately, the Pandat calculations confirm that the equilibrium content of the insoluble Fe-rich phase in this alloy system is consistently higher than that of the Mg_2_Si phase.

### 3.2. Evolution of the Second Phase During Homogenization and Solid Solution Processes

**Figure 7:**
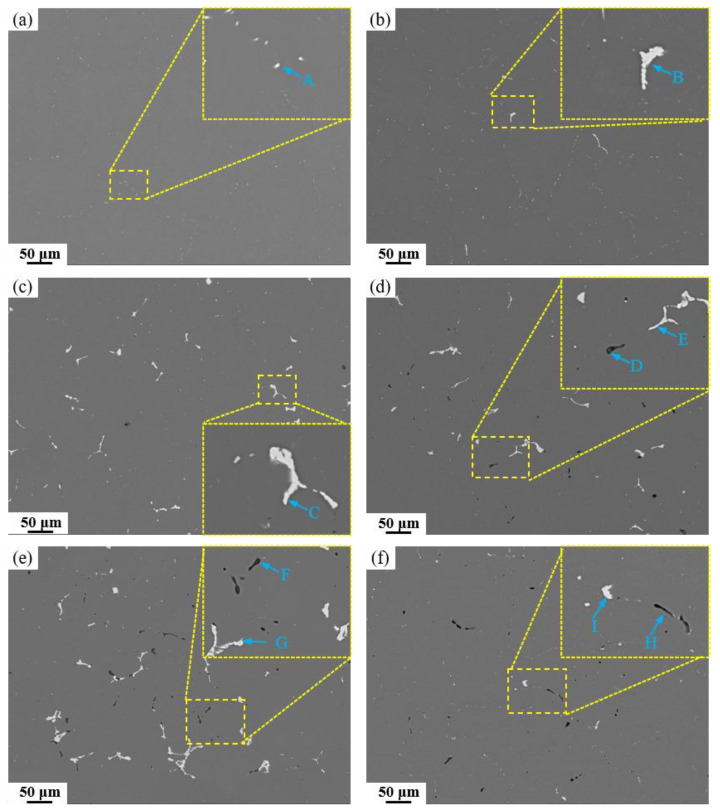
SEM images of alloys with different Fe and Si contents after homogenization: (**a**) Base alloy; (**b**) 0.03Fe 0.02Si; (**c**) 0.15Fe 0.02Si; (**d**) 0.12Fe 0.08Si; (**e**) 0.15Fe 0.12Si; (**f**) 0.03Fe 0.12Si.

Microstructural analysis of the as-cast alloy reveals a characteristic dendritic network with severe segregation, where coarse secondary phases have formed both at grain boundaries and within the grains. The homogenization heat treatment is designed to address this by dissolving these coarse non-equilibrium eutectic phases, thereby reducing elemental microsegregation and achieving a more homogeneous elemental distribution. Based on the endothermic peaks observed in the DSC curve of the as-cast alloys, a three-step homogenization treatment was designed to prevent incipient melting. The first stage aims mainly to facilitate the precipitation of Al_3_Zr dispersoids; the second stage targets the dissolution of the AlZnMgCu phase; and the third stage is intended to dissolve residual Al_2_Cu or Al_2_CuMg phases. Figure 7 displays SEM images of the alloys after homogenization heat treatment, with corresponding EDS analysis of the marked locations provided in Table 3 (the energy-dispersive spectrum can be found in Supplementary Figure S2). The resolvable phases have completely dissolved into the aluminum matrix. The base alloy exhibits the least amount of residual secondary phases, consisting only of dot-like Fe/Si-rich particles approximately 1~2 μm in size located at grain boundaries. As the Fe content increases, both the number and size of Fe-rich phases rise significantly. EDS results confirm that these Fe-rich phases are predominantly Al_7_Cu_2_Fe. In high Si-containing alloys, the Mg_2_Si phase remains undissolved and retains a short rod-like morphology, extensively distributed along grain boundaries. The detection of oxygen in the EDS analysis is attributed to the fact that Mg_2_Si is highly susceptible to oxidation.

**Figure 8:**
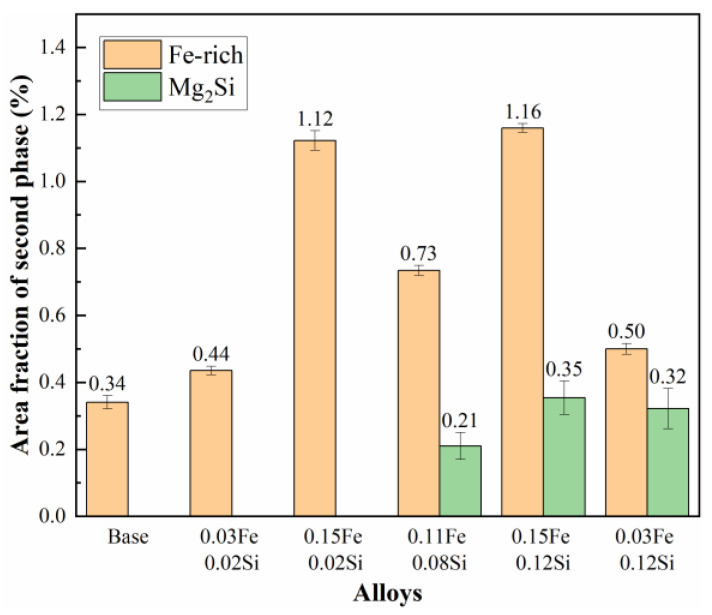
Area fraction of residual secondary phases after homogenization.

Statistical analysis of the residual phases in SEM samples after homogenization was conducted to quantitatively evaluate the effect of Fe and Si contents on impurity phase formation. The area fractions of these residual phases are summarized in Figure 8. The results indicate that the base alloy and the 0.03Fe 0.02Si alloy exhibit relatively low area fractions of Fe-rich phases, measuring 0.34% and 0.44%, respectively. However, as the Fe content increases from 0.03 wt.% to 0.15 wt.%, the area fraction of Fe-rich phases rises markedly from 0.44% to 1.12%. In contrast, when the Si content is maintained at a low level of 0.02 wt.%, the area fraction of the Mg_2_Si phase remains nearly negligible, irrespective of variations in Fe content (from 0.03Fe to 0.15Fe). However, in 0.08 and 0.12 wt.% Si alloys, significant precipitation of the Mg_2_Si phase occurred, with area fractions reaching 0.21% and 0.35%, respectively. These results demonstrate that substantial Mg_2_Si formation is only initiated when the Si content exceeds a critical threshold. Notably, a discernible interaction between Fe and Si elements was observed. In alloys with fixed Fe contents of 0.03 and 0.15 wt.%, increasing the Si content from 0.02% to 0.12% not only promoted Mg_2_Si precipitation but also resulted in a discernible increase in the area fraction of Fe-rich phases by 0.06% and 0.04%, respectively. This synergistic effect is likely associated with the enhanced formation of the ternary α-AlFeSi phase, thereby validating the earlier microstructural observations and thermodynamic simulation results.

**Figure 9:**
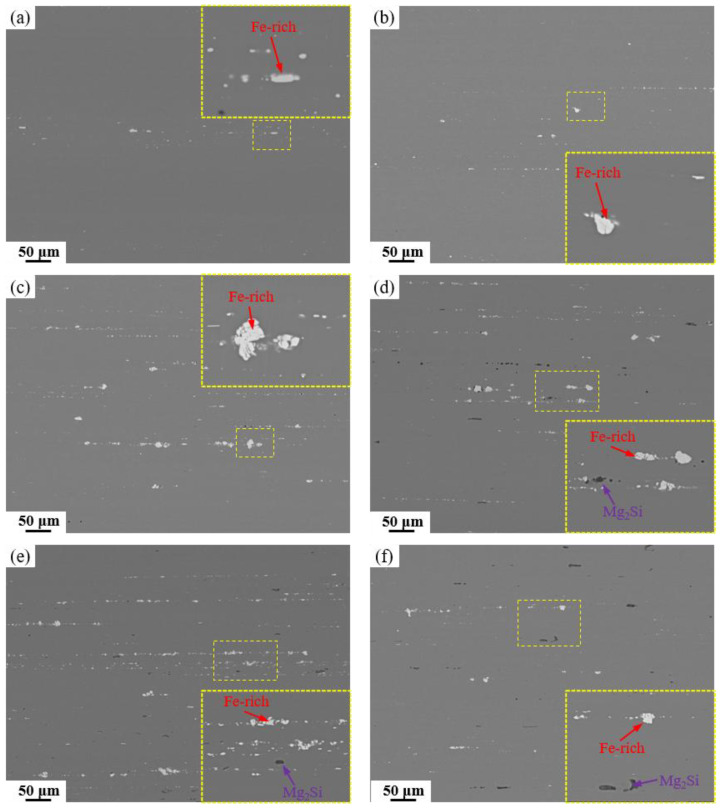
SEM images and locally magnified images of alloys with different Fe and Si contents after solution treatment: (**a**) Base alloy; (**b**) 0.03Fe 0.02Si; (**c**) 0.15Fe 0.02Si; (**d**) 0.12Fe 0.08Si; (**e**) 0.15Fe 0.12Si; (**f**) 0.03Fe 0.12Si.

**Figure 10:**
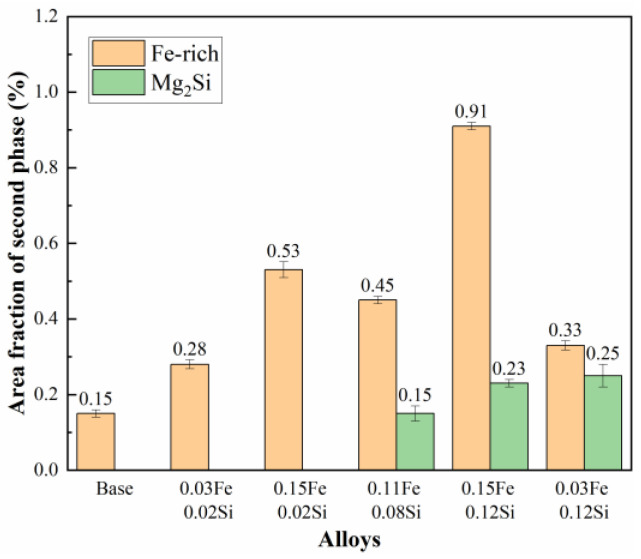
Area fraction of residual secondary phases after solution treatment.

Following homogenization, the alloy was subjected to hot extrusion at 400 °C and subsequent solution treatment at 470 °C/1 h + 480 °C/1 h. The corresponding SEM images in Figure 9 reveal that the originally coarse Fe-rich and Mg_2_Si phases were effectively fractured and refined under intense shear stress during extrusion, leading to their alignment in chain or string-like arrays along the extrusion direction. This fragmentation markedly improved the distribution homogeneity of the secondary phases and mitigated their detrimental cutting effect on the aluminum matrix. Owing to their inherent high hardness and brittleness, the Fe-rich phases experienced more pronounced fragmentation, resulting in the presence of numerous spherical Fe-rich particles at the micrometer scale. Figure 10 summarizes the area fractions of Fe-rich and Mg_2_Si phases after hot extrusion and solution treatment. A significant reduction in the area fractions of both types of second phases is observed across all alloys, which is primarily due to the fragmentation of coarse and continuous secondary phases into finer particles during hot extrusion. This morphological transformation not only improves phase distribution but also converts interconnected phases into discrete particles, leading to an apparent decrease in their measured area fractions in two-dimensional metallographic sections. Nevertheless, in alloys with high Fe and Si contents, considerable amounts of Fe-rich and Mg_2_Si phases persist, with area fractions reaching 0.91% and 0.23%, respectively. The combined deformation and solution treatment effectively converted the detrimental coarse insoluble phases into fine particles while dissolving soluble phases in preparation for subsequent aging, thereby markedly optimizing the alloy’s microstructure.

### 3.3. Mechanical Properties

**Figure 11:**
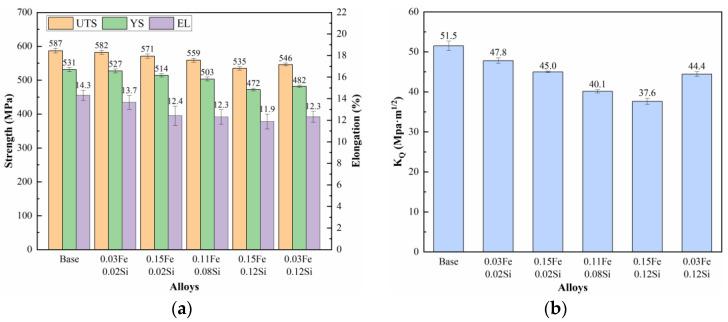
Mechanical properties of alloys with different Fe and Si contents: (**a**) Tensile properties; (**b**) Fracture toughness.

The solution-treated samples were subjected to a 2% pre-stretch deformation followed by aging at 120 °C/4 h + 164 °C/20 h. Figure 11 presents the tensile properties and fracture toughness of the alloys with varying Fe and Si contents. As shown, the base alloy demonstrates the highest strength, elongation, and fracture toughness, while the addition of Fe and Si leads to a general degradation in mechanical performance. When the Si content is fixed at 0.02% wt.%, increasing the Fe content from 0.03% wt.% to 0.15% wt.% results in a reduction in tensile strength by 5 MPa and 16 MPa, a decrease in elongation by 0.6% and 1.9%, and a decline in fracture toughness by 3.7 MPa·m^1/2^ and 6.5 MPa·m^1/2^, respectively, relative to the base alloy. In alloys with a constant Fe content of 0.03% wt.%, raising the Si content from 0.02% wt.% to 0.12% wt.% leads to a more pronounced deterioration: tensile strength drops by 5 MPa and 41 MPa, elongation decreases by 0.6% and 2.0%, and fracture toughness falls by 3.7 MPa·m^1/2^ and 7.1 MPa·m^1/2^, respectively. Similarly, under a fixed Fe content of 0.15%, increasing Si from 0.02% wt.% to 0.12% wt.% leads to even greater degradation, with tensile strength declining by 16 MPa and 52 MPa, elongation reduced by 1.9% and 2.4%, and fracture toughness lowered by 6.5 MPa·m^1/2^ and 13.9 MPa·m^1/2^, respectively. The results indicate that increasing the Fe content leads to a relatively modest reduction in strength but a more pronounced deterioration in fracture toughness. In contrast, elevated Si content results in substantial degradation of strength, ductility, and fracture toughness. The most severe mechanical property deterioration occurs when both Fe and Si are present at high levels, suggesting a synergistic detrimental effect.

**Figure 12:**
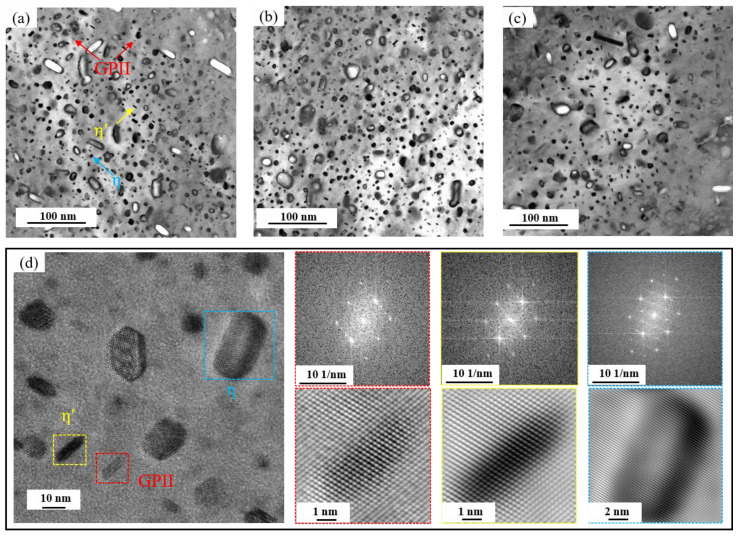
BF and HR-TEM images of alloys along <110>_Al_ zone axis: (**a**,**d**) Base alloy; (**b**) 0.15Fe 0.02Si alloy; (**c**) 0.15Fe 0.12Si alloy.

Figure 12 presents TEM images of the base alloy, 0.15Fe 0.02Si alloy, and 0.15Fe 0.12Si alloy observed along the ⟨110⟩_Al_ zone axis. Coupled with high-resolution TEM characterization of the base alloy, three types of precipitates are identified as the primary strengthening phases: GPII zones, η′, and η phases. The GPII zones, only a few atomic layers thick, remain fully coherent with the Al matrix. The η′ phase, a metastable transition phase transformed from GP zones, exhibits partial coherency with the matrix. In addition, the stable equilibrium η phase is also present, consistent with the over-aged condition of the alloy. All these precipitates consist mainly of Mg and Zn atoms. It is noteworthy that increasing the Fe content alone has limited influence on the volume fraction of strengthening precipitates. In contrast, a further increase in Si content leads to a remarkable decrease in the density of these strengthening phases, resulting in a significant drop in strength.

**Figure 13:**
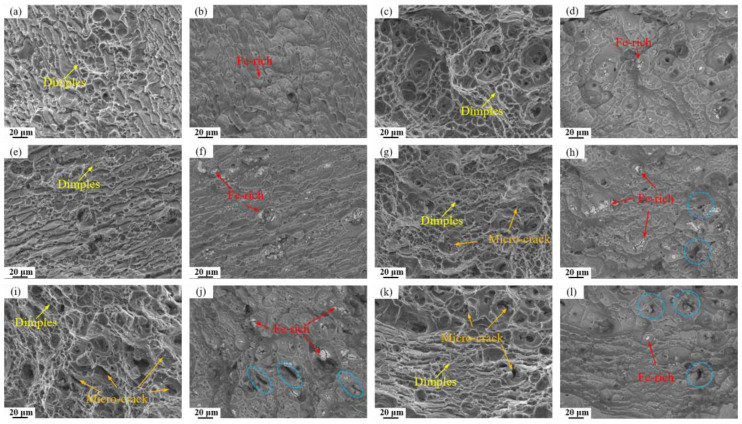
SE and BSE-SEM images in tensile fracture surfaces of alloys: (**a**,**b**) Base alloy; (**c**,**d**) 0.03Fe 0.02Si; (**e**,**f**) 0.15Fe 0.02Si; (**g**,**h**) 0.12Fe 0.08Si; (**i**,**j**) 0.15Fe 0.12Si; (**k**,**l**) 0.03Fe 0.12Si.

Figure 13 displays the fracture surfaces of the investigated alloys observed in both secondary electron (SE) and backscattered electron (BSE) modes. The BSE images clearly indicate an increasing density of Fe-rich particles on the fracture surface of alloy with higher Fe content. Additionally, numerous microcracks associated with detached black Mg_2_Si particles are evident in high Si- containing alloys. SE observations reveal that while all alloys exhibit ductile fracture characteristics with abundant dimples, those containing Fe and Si show significantly more microcracks initiated around impurity second phase. These findings further confirm that the retained Fe-rich and Mg_2_Si phases (blue dash circle) act as stress concentration sites during tensile deformation, promoting microcrack nucleation and ultimately leading to the degradation of mechanical properties.

The above research findings indicate that both Fe and Si impurity elements negatively impact the tensile properties of the alloy, though the extent and mechanism of their effects differ significantly. A combination of mechanical property data and microstructural analysis reveals that increased Si content is the primary factor causing a comprehensive and significant decline in the alloy’s strength, plasticity, and toughness. In contrast, the influence of Fe is relatively weaker and primarily reflects in its detrimental effect on toughness. This difference stems from the distinct properties of the secondary phases formed by each element and their differing impacts on microstructure and deformation processes. As revealed by TEM analysis (Figure 12), the main strengthening precipitates (GPII zones, η′, and η phases) are all composed of Mg and Zn atoms. In the case of Fe, it forms coarse, insoluble intermetallic compounds such as Al_7_Cu_2_Fe. Since these Fe-rich phases remain undissolved during solution treatment, they do not consume the Mg and Zn solute atoms necessary for the nucleation of GP zones or η′ precipitates during aging. As a result, increasing the Fe content alone (e.g., from 0.03% to 0.15%) causes only minor variations in the volume fraction of strengthening phases, leading to a relatively small strength reduction (5 MPa and 16 MPa, respectively), significantly less than that induced by Si. Nevertheless, these hard and brittle Fe-rich particles readily decohere from the matrix or fracture during tensile deformation, acting as potent sites for microcrack initiation [35,36]. This is clearly evidenced in BSE images (Figure 13) by microcracks forming around Fe-rich phases, which accounts for the notable deterioration in elongation and fracture toughness.

In contrast, Si exerts a more profound and multifaceted detrimental effect. Si preferentially combines with Mg to form Mg_2_Si compounds. While some coarse Mg_2_Si particles form during solidification and persist through subsequent thermal processing, thereby reducing the concentration of Mg atoms essential for precipitating key strengthening phases (GPII zones and η’ phases) during aging. This mechanism is directly confirmed by TEM analysis (Figure 12), which reveals a substantial decrease in strengthening phase density with increasing Si content. The consequent weakening of precipitation strengthening leads to significant strength loss, as evidenced by the 41 MPa reduction in the 0.03Fe 0.12Si alloy. Furthermore, BSE images of fracture surfaces show that Mg_2_Si particle detachment generates additional microcracks, further aggravating the deterioration of both ductility and fracture toughness.

Therefore, Fe primarily affects toughness and plasticity by introducing crack initiation sites, while having limited impact on the quantity of strengthening phases. While Si consumes the key alloying element Mg, it simultaneously causes comprehensive and more severe damage to strength, plasticity, and toughness. When both Fe and Si contents are elevated, these two deterioration mechanisms exhibit synergistic effects. Fe-rich phases and Mg_2_Si phases act as stress concentration points, significantly promoting microcrack initiation and propagation, leading to the most severe degradation of the alloy’s mechanical properties [37,38,39].

### 3.4. Exfoliation Corrosion Performance

**Figure 14:**
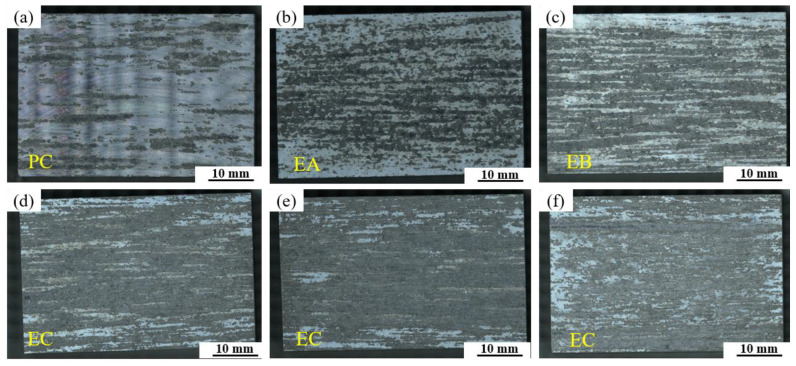
Macrostructural images of exfoliation corrosion surfaces for alloys with different Fe and Si contents: (**a**) Base alloy; (**b**) 0.03Fe 0.02Si; (**c**) 0.15Fe 0.02Si; (**d**) 0.12Fe 0.08Si; (**e**) 0.15Fe 0.12Si; (**f**) 0.03Fe 0.12Si.

Figure 14 presents macrographs of exfoliation corrosion surfaces for alloys with different Fe and Si contents. The base alloy exhibits only slight, sparse pitting attacks or fine filiform corrosion marks, corresponding to a PC corrosion grade. As the Fe and Si contents increase individually, the alloys develop more severe corrosion cracks and localized exfoliation, with the corrosion severity increasing markedly. The most extensive corrosion damage is observed in the high Fe and Si-containing alloys (0.12Fe 0.08Si and 0.15Fe 0.12Si). When both Fe and Si are present at elevated levels, they induce the most detrimental synergistic effect, substantially deteriorating the alloy’s resistance to exfoliation corrosion.

**Figure 15:**
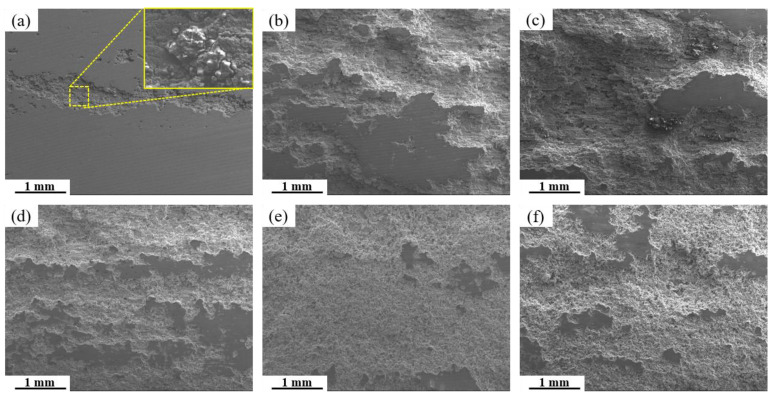
SEM images of exfoliation corrosion surfaces for alloys with different Fe and Si contents: (**a**) Base alloy; (**b**) 0.03Fe 0.02Si; (**c**) 0.15Fe 0.02Si; (**d**) 0.12Fe 0.08Si; (**e**) 0.15Fe 0.12Si; (**f**) 0.03Fe 0.12Si.

**Figure 16:**
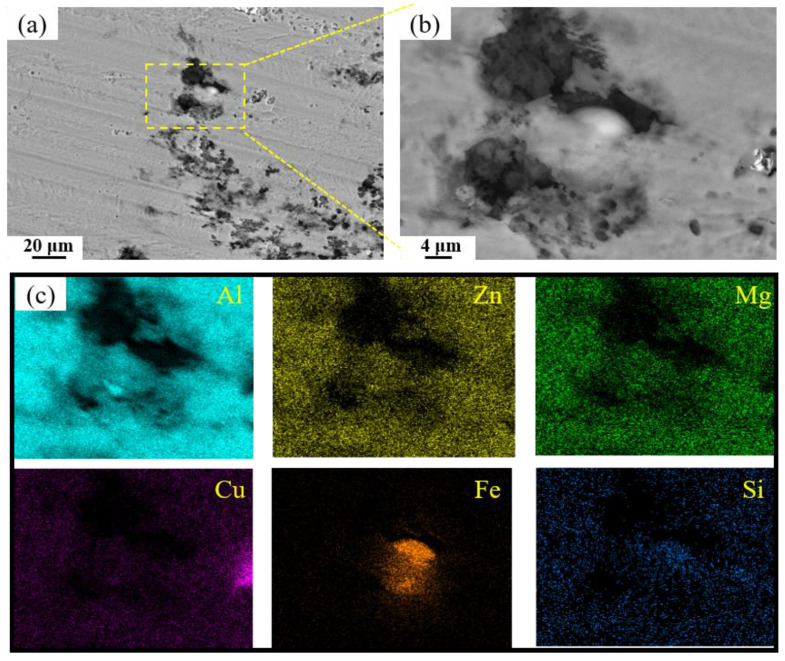
SEM images of exfoliation corrosion surfaces for 0.12Fe 0.08Si alloys: (**a**,**b**) SEM; (**c**) elemental mapping.

Figure 15 presents SEM micrographs of the exfoliation corrosion surface for alloys with varying Fe and Si contents. In the base alloy, localized pitting and interconnected network-like cracks are visible, accompanied by the accumulation of white powdered corrosion products within cracks and exfoliated gaps. With increasing Fe and Si contents, the severity of exfoliation corrosion is markedly exacerbated. A comparison between the 0.15Fe 0.02Si and 0.03Fe 0.12Si alloys indicates that higher Si content substantially impairs exfoliation corrosion resistance. The most severe corrosion damage occurs in the 0.15Fe 0.12Si alloy, where the surface is almost entirely corroded. The simultaneous elevation of both Fe and Si contents leads to the most pronounced deterioration in exfoliation corrosion resistance. Figure 16 shows the high-magnification SEM morphology and elemental mapping of the corroded surface of the 0.12Fe 0.08Si alloy. Due to the prolonged corrosion time, the second phases on the alloy surface have almost completely spalled off. A small amount of Fe-rich impurity phases remains within some corrosion pits, further indicating that such impurity phases are prone to initiating corrosion.

The findings presented above underscore the substantial impact of Fe and Si contents on the exfoliation corrosion resistance of the 7050 aluminum alloy. Macroscopic examination clearly indicates that the base alloy (with low Fe and Si levels) possesses the highest corrosion resistance, displaying only minor pitting and filiform corrosion, corresponding to a PC grade. However, the introduction of Fe and Si, especially when both are added concurrently, leads to a sharp transition in corrosion morphology from localized pitting and filiform attack to severe exfoliation and interconnected network cracking. This behavior reflects a pronounced synergistic deterioration effect between Fe and Si on the alloy’s exfoliation corrosion performance. As detrimental impurity elements, Fe and Si tend to form intermetallic compounds that act as cathodic phases relative to the aluminum matrix. Within the resulting micro-galvanic couples, these compounds accelerate anodic dissolution of the adjacent aluminum matrix. More critically, when distributed along grain boundaries, they disrupt the continuity of the protective surface oxide film, thereby providing easy access for corrosive agents and facilitating the formation of anodic zones at grain boundaries. These processes collectively intensify the initiation and propagation of exfoliation corrosion [40,41,42].

## 4. Conclusions

In the present work, the effect of Fe and Si content on microstructure, mechanical properties, and corrosion resistance was studied in 7050 alloy. The main conclusions are as follows:

(1) Impurity elements Fe and Si induce the formation of insoluble Fe-rich phases (e.g., Al_7_Cu_2_Fe, Al_6_Fe, and α-AlFeSi) and Mg_2_Si phases in the alloy, respectively. These phases persist extensively along grain boundaries after homogenization and solution heat treatments, with their area fractions increasing notably with higher Fe and Si contents.
(2) The concurrent addition of Fe and Si results in a severe synergistic deterioration of mechanical properties. The most pronounced degradation occurs at 0.15 wt.% Fe and 0.12 wt.% Si, where the alloy exhibits a tensile strength reduction of 52 MPa, a loss of 2.4% in elongation, and a drastic decrease of 13.9 MPa·m^1/2^ in fracture toughness relative to the base alloy.
(3) Si has a more profound negative impact on mechanical properties than Fe. Fe primarily reduces ductility and fracture toughness through Fe-rich phases that initiate microcracks, with minimal effect on strength. Si not only forms brittle Mg_2_Si phases that impair toughness but also significantly reduces the quantity of strengthened phases (GPII zone, η’ phase) by consuming Mg elements in the matrix, leading to a comprehensive and severe decline in strength, plasticity, and toughness.
(4) Fe and Si impurities markedly degrade the exfoliation corrosion resistance of the alloy. The Fe-rich and Mg_2_Si phases, serving as active cathodes, establish micro-galvanic couples with the aluminum matrix. This accelerates the dissolution of the anodic zone around grain boundaries, thereby strongly inducing and accelerating exfoliation corrosion.

## Figures and Tables

**Table 1:** Chemical composition for investigated alloy(wt.%).

Alloys	Cu	Mg	Zn	Zr	Fe	Si	Al
Base	2.28	2.26	6.21	0.10	0.0034	0.0057	Bal.
0.03Fe 0.02Si	2.22	2.34	6.15	0.11	0.035	0.022	Bal.
0.15Fe 0.02Si	2.32	2.35	6.24	0.12	0.16	0.027	Bal.
0.12Fe 0.08Si	2.26	2.33	6.14	0.12	0.13	0.076	Bal.
0.15Fe 0.12Si	2.25	2.31	6.25	0.12	0.16	0.12	Bal.
0.03Fe 0.12Si	2.24	2.24	6.14	0.12	0.038	0.12	Bal.

**Table 2:** EDS analysis from the points marked in Figure 3 (at.%).

Alloys		Al	Zn	Mg	Cu	Fe	Si	O	Phase
Base	A	64.55	9.85	16.38	9.11		0.11		AlZnMgCu
0.03Fe 0.02Si	B	71.14	2.47	1.94	24.36	0.09			Al_2_Cu
	C	43.19	15.37	26.33	14.82	0.07	0.21		AlZnMgCu
0.15Fe 0.02Si	D	84.05	1.38	0.56	1.68	13.49	0.22		Fe-rich
0.12Fe 0.08Si	E	78.34	2.07	0.71	5.89	10.72	2.27		Fe-rich
0.15Fe 0.12Si	F	81.90	2.91	1.18	1.57	12.02	0.43		Fe-rich
0.03Fe 0.12Si	G	19.10	0.38	34.14	0.14	0.08	21.78	24.38	Mg2Si

**Table 3:** EDS analysis of the second phases in alloys after homogenization (at.%).

Alloys		Al	Zn	Mg	Cu	Fe	Si	O	Phase
Base	A	83.55	1.41	8.80	5.66	0.13	0.46		Fe/Si-rich
0.03Fe 0.02Si	B	74.16	0.69	1.92	15.07	7.59	0.57		Fe-rich
0.15Fe 0.02Si	C	75.62	0.42	2.14	14.04	7.28	0.49		Fe-rich
0.12Fe 0.08Si	D	32.65	0.65	14.92	0.21	0.07	19.73	31.77	Mg_2_Si
	E	73.23	0.49	2.66	15.15	7.84	0.65		Fe-rich
0.15Fe 0.12Si	F	34.24	0.50	37.05	0.15	0.06	16.55	11.45	Mg_2_Si
	G	74.31	0.62	2.40	14.48	7.51	0.67		Fe-rich
0.03Fe 0.12Si	H	18.70	0.43	29.07	0.24	0.10	18.86	32.6	Mg_2_Si
	I	74.16	0.62	1.95	15.05	7.62	0.60		Fe-rich

## Data Availability

The original contributions presented in the study are included in the article/Supplementary Material, further inquiries can be directed to the corresponding authors.

## Supplementary Materials

The following supporting information can be downloaded at: https://www.mdpi.com/article/10.3390/ma19010135/s1, Figure S1: XRD; Figure S2: The energy-dispersive spectrum of the marked point in Figure 3; Figure S3: The energy-dispersive spectrum of the marked point in Figure 7.
