# Supplementary material for: Effect of Fe and Si Content on Microstructure, Mechanical Properties, and Corrosion Resistance of 7050 Alloy
Source: Materials (Basel). 2025 Dec 30;19(1):135. doi: 10.3390/ma19010135 (PMC12787006; doi:10.3390/ma19010135)
Supplement: Supplementary file 1 [file materials-19-00135-s001.zip › materials-4042377-supplementary.pdf]

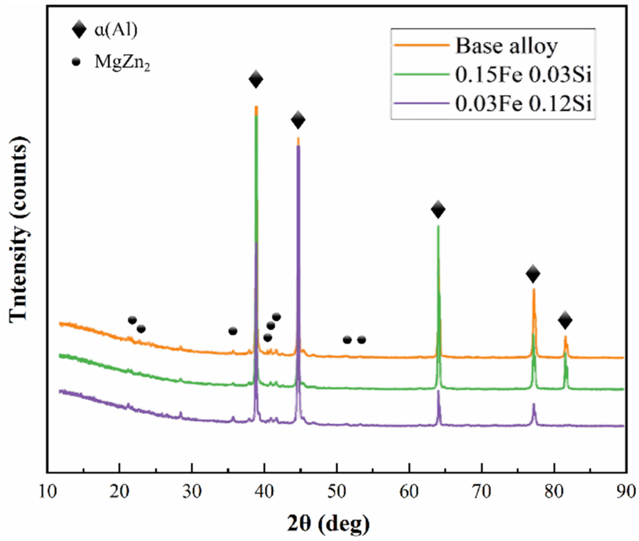

Figure S1 XRD

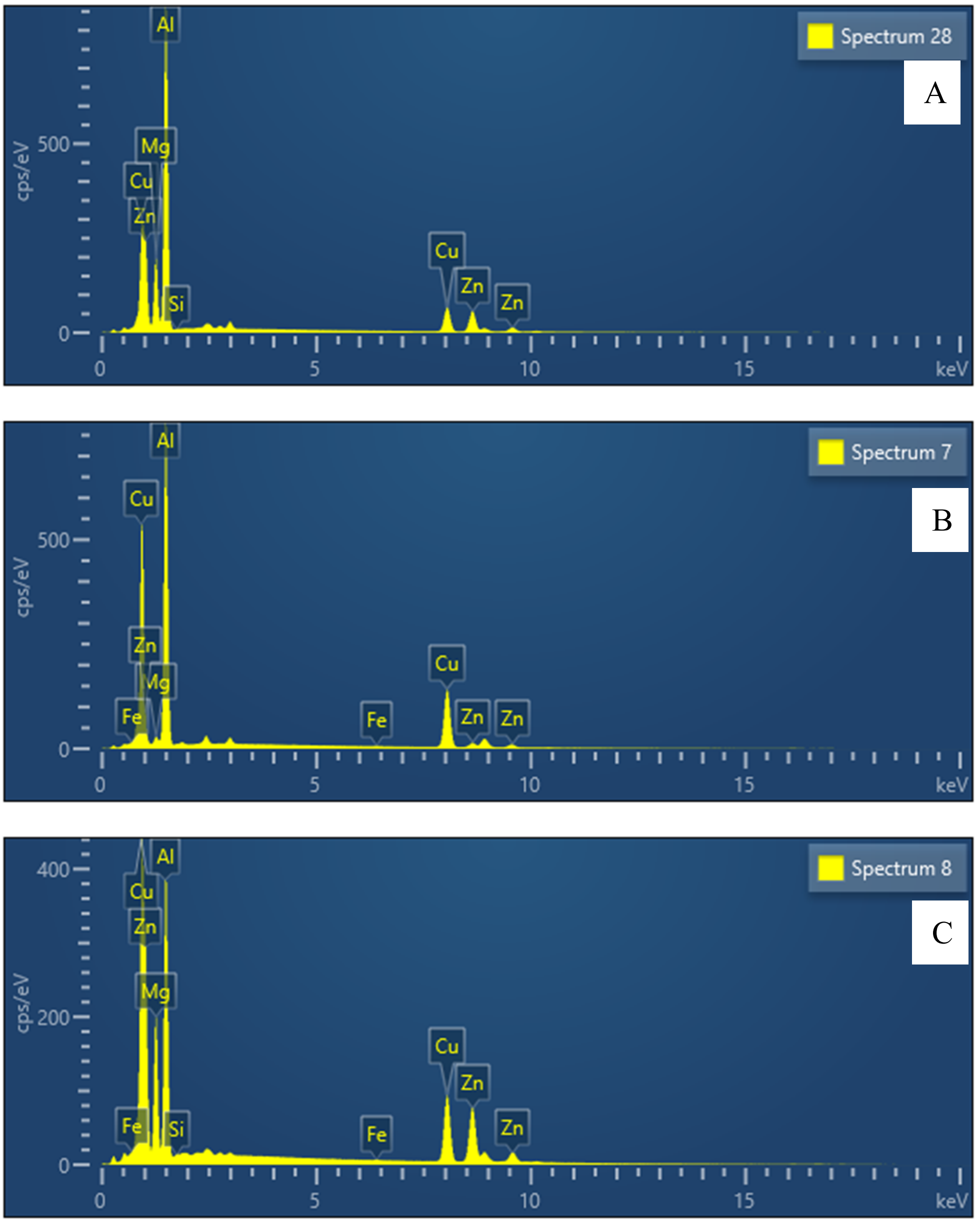

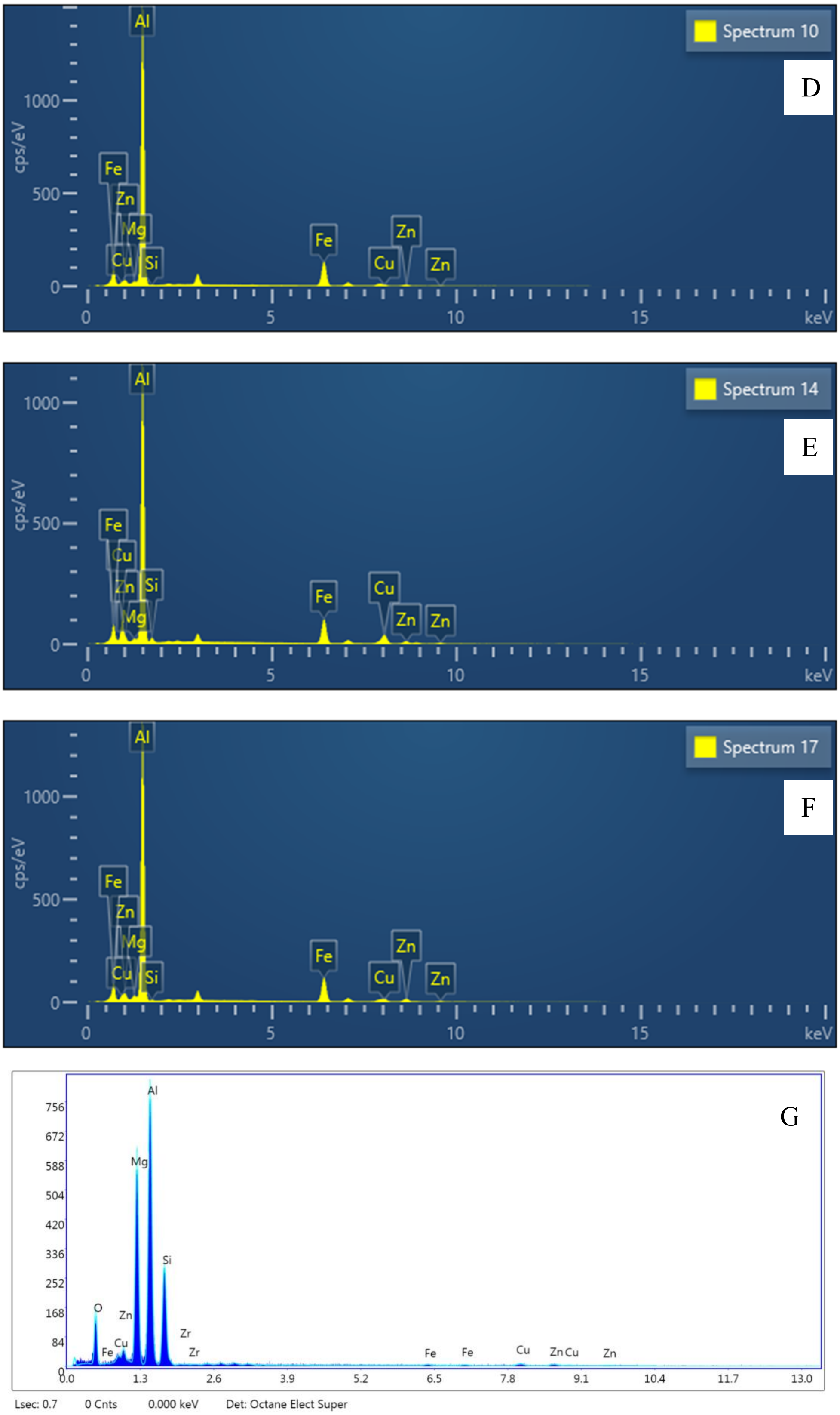

Figure S2 The energy-dispersive spectrum of the marked point in Figure 3.

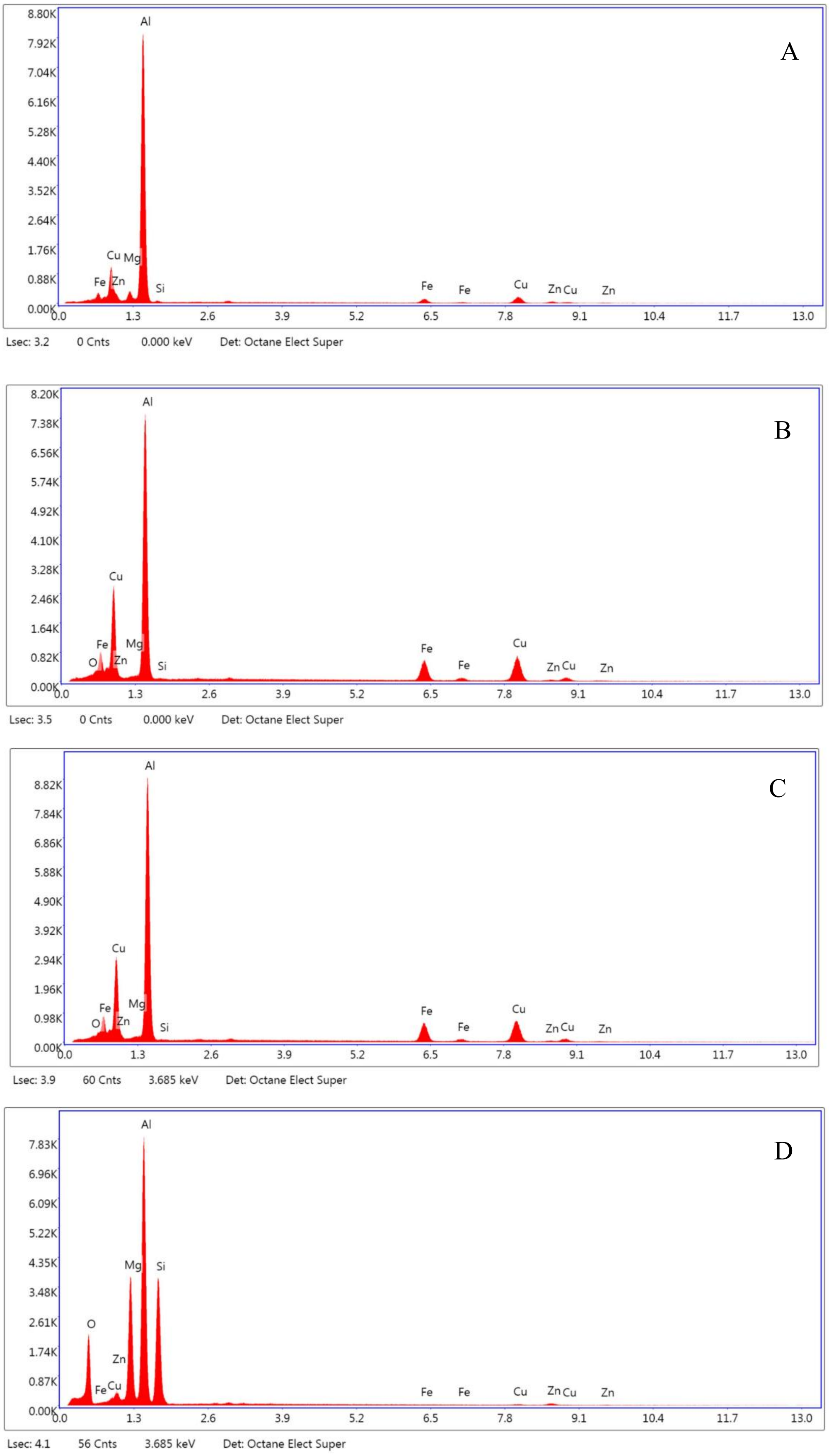
A
Al
Cu Mg
Fe Zn
Si
Fe
Fe
Cu
Zn Cu
Zn
8.80K
7.92K
7.04K
6.16K
5.28K
4.40K
3.52K
2.64K
1.76K
0.88K
0.00K
0.0 1.3 2.6 3.9 5.2 6.5 7.8 9.1 10.4 11.7 13.0
Lsec: 3.2 0 Cnts 0.000 keV Det: Octane Elect Super
B
Al
Cu
Fe
Mg
O
Zn
Si
Fe
Fe
Cu
Zn Cu
Zn
8.20K
7.38K
6.56K
5.74K
4.92K
4.10K
3.28K
2.46K
1.64K
0.82K
0.00K
0.0 1.3 2.6 3.9 5.2 6.5 7.8 9.1 10.4 11.7 13.0
Lsec: 3.5 0 Cnts 0.000 keV Det: Octane Elect Super
C
Al
Cu
Fe
Mg
O
Zn
Si
Fe
Fe
Cu
Zn Cu
Zn
8.82K
7.84K
6.86K
5.88K
4.90K
3.92K
2.94K
1.96K
0.98K
0.00K
0.0 1.3 2.6 3.9 5.2 6.5 7.8 9.1 10.4 11.7 13.0
Lsec: 3.9 60 Cnts 3.685 keV Det: Octane Elect Super
D
Al
Mg
Si
O
Zn
Fe Cu
Fe
Fe
Cu
Zn Cu
Zn
7.83K
6.96K
6.09K
5.22K
4.35K
3.48K
2.61K
1.74K
0.87K
0.00K
0.0 1.3 2.6 3.9 5.2 6.5 7.8 9.1 10.4 11.7 13.0
Lsec: 4.1 56 Cnts 3.685 keV Det: Octane Elect Super

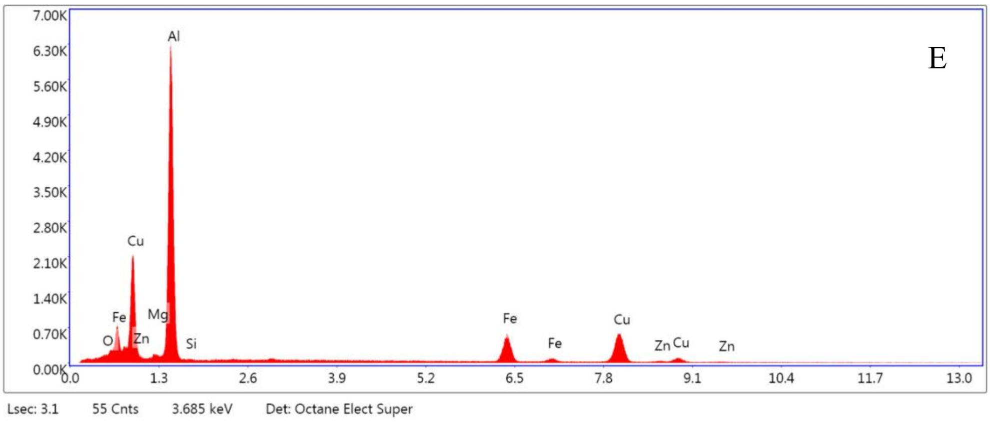
E
Al
Cu
Fe
Mg
O
Zn
Si
Fe
Fe
Cu
Zn Cu
Zn
7.00K
6.30K
5.60K
4.90K
4.20K
3.50K
2.80K
2.10K
1.40K
0.70K
0.00K
0.0
1.3
2.6
3.9
5.2
6.5
7.8
9.1
10.4
11.7
13.0
Lsec: 3.1 55 Cnts 3.685 keV Det: Octane Elect Super

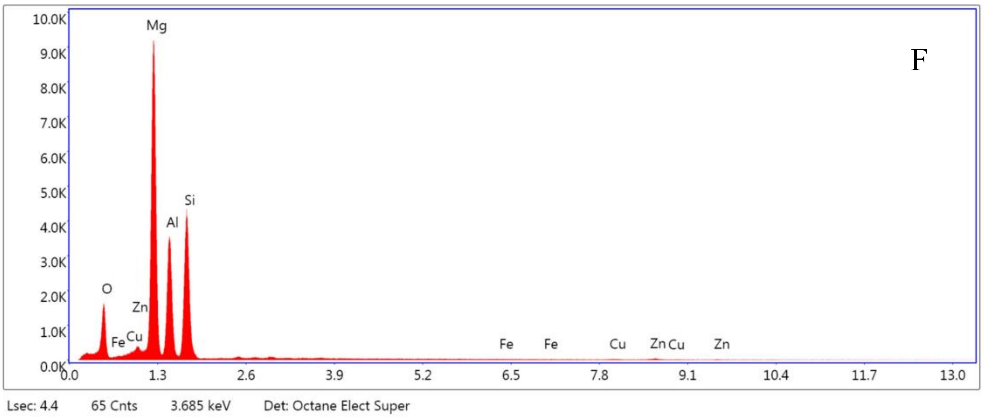
F
Mg
Si
Al
O
Zn
Fe
Cu
Fe
Fe
Cu
Zn Cu
Zn
10.0K
9.0K
8.0K
7.0K
6.0K
5.0K
4.0K
3.0K
2.0K
1.0K
0.0K
0.0
1.3
2.6
3.9
5.2
6.5
7.8
9.1
10.4
11.7
13.0
Lsec: 4.4 65 Cnts 3.685 keV Det: Octane Elect Super

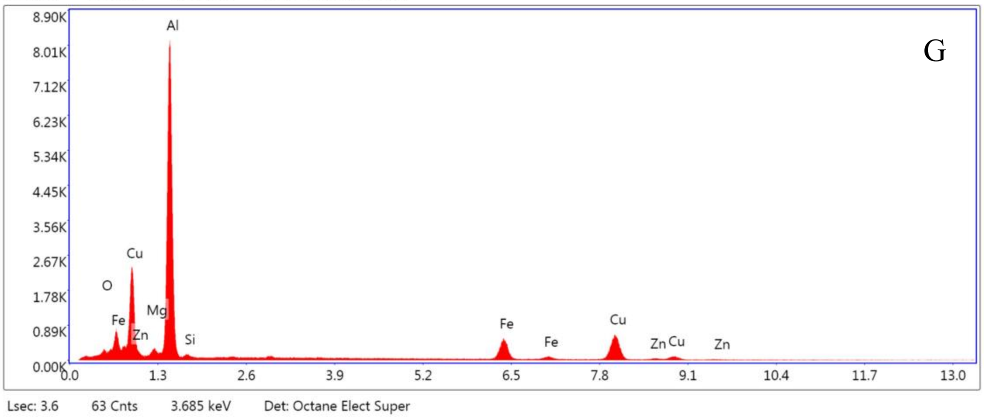
G
Al
Cu
O
Fe
Mg
Zn
Si
Fe
Fe
Cu
Zn Cu
Zn
8.90K
8.01K
7.12K
6.23K
5.34K
4.45K
3.56K
2.67K
1.78K
0.89K
0.00K
0.0
1.3
2.6
3.9
5.2
6.5
7.8
9.1
10.4
11.7
13.0
Lsec: 3.6 63 Cnts 3.685 keV Det: Octane Elect Super

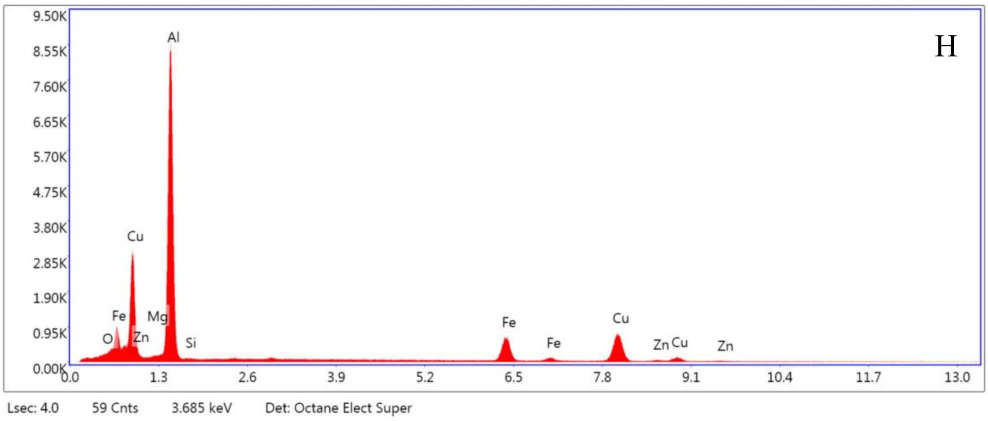
H
Al
Cu
Fe
Mg
O
Zn
Si
Fe
Fe
Cu
Zn Cu
Zn
9.50K
8.55K
7.60K
6.65K
5.70K
4.75K
3.80K
2.85K
1.90K
0.95K
0.00K
0.0
1.3
2.6
3.9
5.2
6.5
7.8
9.1
10.4
11.7
13.0
Lsec: 4.0 59 Cnts 3.685 keV Det: Octane Elect Super

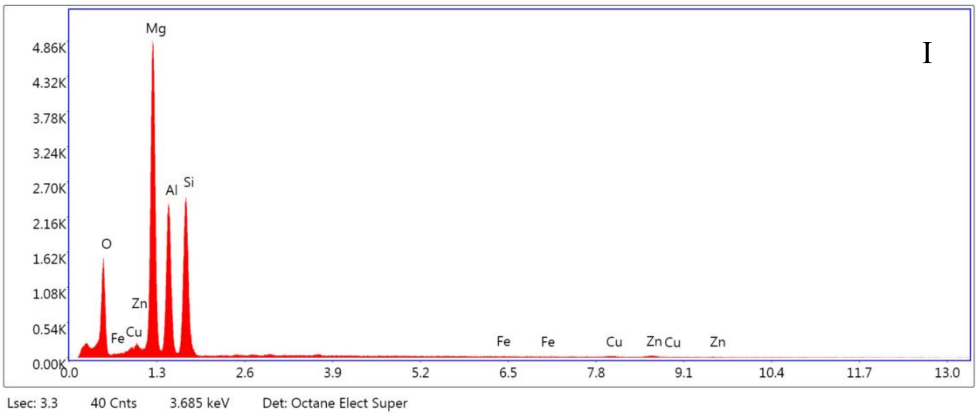

Figure S3 The energy-dispersive spectrum of the marked point in Figure 7.
